# P-555. Temporal Trends in Rates of Hospitalized Pneumonia among US Adults and Residual Disparities by Race and Socioeconomic Status

**DOI:** 10.1093/ofid/ofaf695.770

**Published:** 2026-01-11

**Authors:** Ahuva Averin, Mark Rozenbaum, Derek Weycker, Rotem Lapidot, Amanda C Miles, Maria J Tort, Jeffrey T Vietri, Alexander Lonshteyn, Stephen I Pelton

**Affiliations:** Avalere Health, Boston, Massachusetts; Pfizer Inc., Randstad, Noord-Holland, Netherlands; Avalere Health, Boston, Massachusetts; Boston Medical Center, Brookline, Massachusetts; Pfizer, New York, NY; Pfizer, Inc, Collegeville, Pennsylvania; Pfizer, Inc., Collegeville, Pennsylvania; Avalere Health, Boston, Massachusetts; Boston Medical Center, Brookline, Massachusetts

## Abstract

**Background:**

Following the introduction of 13-valent pneumococcal conjugate vaccine (PCV13) for children in the United States (US) in 2010, rates of invasive pneumococcal disease (IPD) among both children and adults declined substantially. The present study examined temporal trends in rates of hospitalized pneumonia (PNE) among adults by age as well as by race and socioeconomic status within age-specific subgroups.

**Figure:**
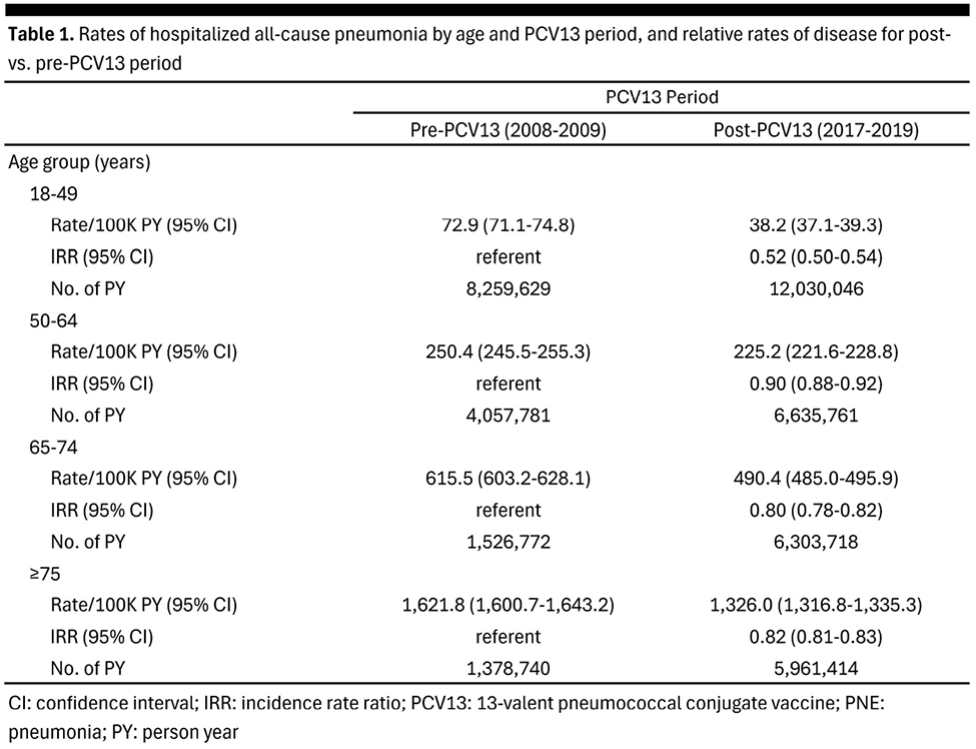

**Figure:**
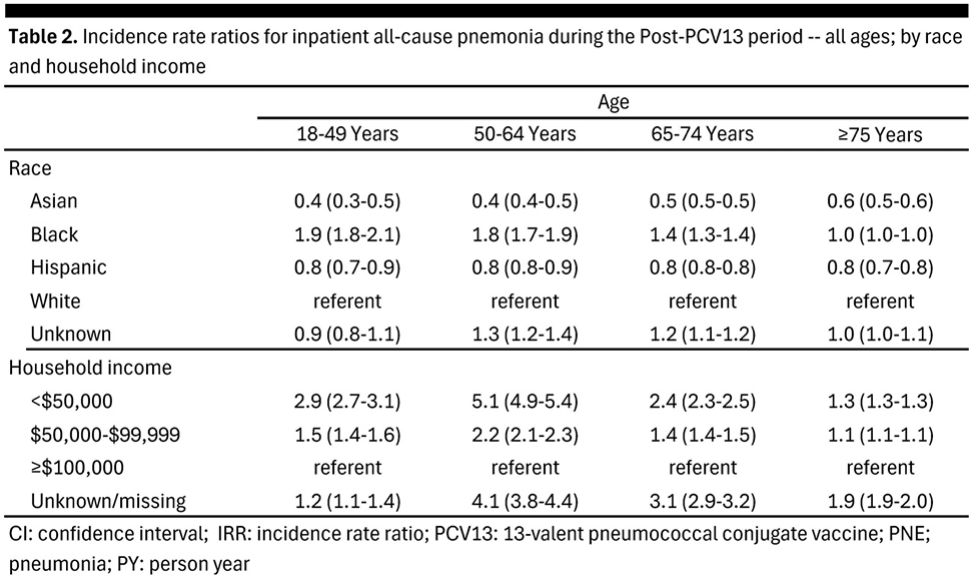

**Methods:**

We used the Optum Clinformatics Datamart to calculate incidence (per 100K patient-years [PY]) of hospitalized all-cause PNE (AC-PNE) among adults aged ≥ 18 years during the 2008-2009 (pre-PCV13) and 2017-2019 (post-PCV13) periods, respectively. Rates were estimated within age groups (18-49, 50-64, 65-74, ≥ 75y) as well as by race (White, Hispanic, Asian, Black) and household income (< $50,000, $50,000-$99,999, ≥ $100,000). Incidence rate ratios (IRRs) comparing AC-PNE during the post-PCV13 period by race and household income were also calculated.

**Results:**

AC-PNE hospitalization rates (per 100K PY) decreased from the pre-PCV13 period to the post-PCV13 period across all ages: 18-49y, 73 to 38 (48% reduction); 50-64y, 250 to 225 (10%); 65-74y, 616 to 490 (20%); ≥ 75y, 1,622 to 1,326 (18%) (Table 1). Similar reductions were observed within age groups across racial and income-based strata. Rates of hospitalized AC-PNE during the post-PCV13 period were higher (generally) for Blacks (IRR [range]: 1.0-1.9), and lower for Asians (IRR [range]: 0.4-0.5) and Hispanics (IRR: 0.8), relative to Whites (Table 2). Hospitalized AC-PNE rates during the post-PCV13 period were higher among adults with annual household income < $50,000 (IRR [range]: 1.3-5.1) and $50,000-$99,999 (IRR [range]: 1.1-2.2), versus those with household income ≥ $100,000.

**Conclusion:**

Rates of hospitalized AC-PNE among adults decreased during the decade following the introduction of PCV13 in the US, however disparities in disease rates defined by race and socioeconomic status persist.

**Disclosures:**

Ahuva Averin, MPP, Pfizer Inc: Grant/Research Support Mark Rozenbaum, PhD, M.B.A., Pfizer: Stocks/Bonds (Public Company) Derek Weycker, Ph.D., Pfizer Inc.: Grant/Research Support Rotem Lapidot, MD, MSCI, Merck: Advisor/Consultant|Merck: Grant/Research Support|Merck: Honoraria|Pfizer: Advisor/Consultant|Pfizer: Grant/Research Support|Pfizer: Honoraria Amanda C. Miles, MPH, Pfizer: Employee of Pfizer Inc.|Pfizer: Stocks/Bonds (Public Company) Maria J. Tort, PhD, Pfizer, Inc: Stocks/Bonds (Public Company) Jeffrey T. Vietri, PhD, Pfizer Inc: Employment|Pfizer Inc: Stocks/Bonds (Public Company) Alexander Lonshteyn, PhD, Pfizer: Grant/Research Support Stephen I. Pelton, MD, CSL Seqirus: Advisor/Consultant|GSK: Grant/Research Support|GSK: Honoraria|Merck Vaccines: Grant/Research Support|Merck Vaccines: Honoraria|Pfizer, Inc.: Grant/Research Support|Pfizer, Inc.: Honoraria|Sanofi: Honoraria|Sanofi: DSMB, Adjudicator for RSV vaccine trial

